# The Role of SBI2/ALG12/EBS4 in the Regulation of Endoplasmic Reticulum-Associated Degradation (ERAD) Studied by a Null Allele

**DOI:** 10.3390/ijms23105811

**Published:** 2022-05-22

**Authors:** Xiaoxia Sun, Di Zhao, Khawar Ali, Yumeng Zhu, Guang Wu, Guishuang Li

**Affiliations:** College of Life Sciences, Shaanxi Normal University, Xi’an 710119, China; xiaoxiasun@snnu.edu.cn (X.S.); shengkezhaodi2022@163.com (D.Z.); aali@snnu.edu.cn (K.A.); zymeng0214@163.com (Y.Z.)

**Keywords:** SBI2/ALG12/EBS4, N-glycosylation, BRI1, stress response, forward genetics

## Abstract

Redundancy and lethality is a long-standing problem in genetics but generating minimal and lethal phenotypes in the knockouts of the same gene by different approaches drives this problem to a new high. In Asn (N)-linked glycosylation, a complex and ubiquitous cotranslational and post-translational protein modification required for the transfer of correctly folded proteins and endoplasmic reticulum-associated degradation (ERAD) of misfolded proteins, ALG12 (EBS4) is an α 1, 6-mannosyltransferase catalyzing a mannose into Glc_3_Man_9_GlcNAc_2_. In *Arabidopsis*, T-DNA knockout *alg12-T* is lethal while likely *ebs4* null mutants isolated by forward genetics are most healthy as weak alleles, perplexing researchers and demanding further investigations. Here, we isolated a true null allele, *sbi2*, with the W258Stop mutation in ALG12/EBS4. *sbi2* restored the sensitivity of brassinosteroid receptor mutants *bri1-5*, *bri1-9*, and *bri1-235* with ER-trapped BRI1 to brassinosteroids. Furthermore, *sbi2* maturated earlier than the wild-type. Moreover, concomitant with impaired and misfolded proteins accumulated in the ER, *sbi2* had higher sensitivity to tunicamycin and salt than the wild-type. Our findings thus clarify the role of SBI2/ALG12/EBS4 in the regulation of the ERAD of misfolded glycoproteins, and plant growth and stress response. Further, our study advocates the necessity and importance of using multiple approaches to validate genetics study.

## 1. Introduction

Protein asparagine-linked glycosylation (N-glycosylation) is an essential modification reaction that occurs widely in archaebacteria, a subset of eubacteria, and most of the eukaryotes. N-glycans play a pivotal role in many diverse biological processes including the folding of polypeptides, transport, sorting, and endoplasmic reticulum-associated degradation (ERAD) [1,2,3,4,5]. Most newly synthesized secretory and membrane proteins require N-glycosylation. The process of N-glycosylation starts on the cytoplasmic side of the endoplasmic reticulum (ER) membrane, where the two acetylglucosamine (GlcNAc) and five mannoses (Man) residues are first added to the lipid carrier (dolichylphosphate) yielding Dol-PP-Man_5_GlcNAc_2_, then the synthesized Dol-PP-Man_5_GlcNAc_2_ chain is translocated into the lumen of the ER. Four more mannoses, including a single α1,3 Man, two α1,2 Man, and an α1,6 Man, residues are sequentially added by three α-mannosyltransferases, ALG3, ALG9, and ALG12, respectively. Further elongation of the oligosaccharide is completed by the successive addition of three glucose residues to the lower branch, which are catalyzed sequentially by three other α-glucosyltransferases, ALG6, ALG8, and ALG10. The fully assembled Glc_3_Man_9_GlcNAc_2_ oligosaccharide is then transferred to the selected asparagine residues within the specific sequence motif Asn-X-Ser/Thr of newly synthesized proteins by the enzyme oligosaccharyltransferase (OST) complex (where X indicates any amino acid except proline while Ser/Thr denotes serine/threonine residues) [6,7,8,9,10]. After the rapid removal of the two glucose residues by α-glucosidases I and II in the ER, the monoglucosylated Glc_1_Man_9_GlcNAc_2_ will interact with the two ER-resident lectins, calnexin and calreticulin (CNX and CRT, respectively). A maturing glycoprotein is liberated from CNX/CRT, thus terminating its folding process in the ER. If the glycoproteins are not properly folded, they will be recognized and reglucosylated by UDP-glucose: glycoprotein glucosyltransferase (UGGT) and undergo additional rounds of the CNX/CRT cycle until the protein are fully complete and mature. The glycoproteins that fail terminally to acquire their native conformations are retained in the ER and eventually are selected for a unique degradative mechanism known as the ERAD mechanism [11]. Significant progress has been made regarding N-glycosylation in plants. For example, the loss of these key enzymes ALG3 [12,13], ALG9 [14], ALG12 [8,15], ALG10 [16], and ALG11 [17] leads to the incomplete synthesis of oligosaccharide precursors, resulting in the reduced glycosylation efficiency of proteins.

Brassinosteroids (BRs), as steroidal hormones in plants, regulate every aspect of plant growth and development. BRs play roles in a diverse range of plant development processes, such as cell elongation, xylem differentiation, fertility, and flowering time. BRs are mainly perceived at the plasma membrane by brassinosteroid insensitive 1 (BRI1), which constitutes a leucine-rich repeat (LRR) extracellular domain (ECD), a transmembrane domain (TMD), and a cytoplasmic kinase domain (KD) with serine/threonine or tyrosine specificity. BR binding to BRI1 promotes its dimerization with its coreceptor BAK1, which then initiates a phosphorylation-mediated signaling cascade. The mutants in the BR biosynthesis or signaling pathway result in a characteristic set of defective phenotypes, including dwarfism, de-etiolated cotyledon, and delayed flowering in *Arabidopsis* [18,19]. Like the vast majority of the secretory and plasma membrane proteins, BRI1 is synthesized and folded in the ER and fully modified in the Golgi complex, which finally moves to the cell surface as a functional receptor. Incomplete or misfolded proteins are retained in the ER to initiate their refolding or to be destroyed via the ERAD pathway [20,21]. The previous studies have shown several ER-retained *bri1* alleles, *bri1-5*, *bri1-235* and *bri1-9*, carrying Cys69Tyr [22], Ser156Phe [23], and Ser662Phe mutation in the extracellular domain of BRI1, respectively [8]. These ER-retained *bri1* mutants offer an additional opportunity to discover how BRI1 is precisely regulated to control BR’s functions.

Genetics study is one of the most effective approaches in life sciences. In fact, much of the knowledge of BR receptors regulated by ERAD has been uncovered through genetics study [8,14,24,25,26,27,28,29,30]. However, one of the fundamental problems is genetic redundancy, in which knocking out a single gene results in no phenotype [30,31]. The other side of the problem is gene essentiality, in which knocking out a single gene is lethal [15]. The former problem is usually resolved by knockouts of all of the duplicated genes while the latter problem can only be circumvented by isolating a weak allele. However, as the study advances, new problems arise such that knockout or knockdown of the same gene by different approaches generates different phenotypes.

Forward genetics is a conventional and inexpensive method, while reverse genetics is a more direct and recent approach. Interestingly, in some occasions, different phenotypes are produced in the knockout of the same gene by different methods [32]. For example, T-DNA insertion might create a null and lethal mutation while some mutants did not display any obvious phenotypic defects obtained by CRISPR-Cas9 or transfer DNA (T-DNA) insertion technology. For decades, AUXIN BINDING PROTEIN 1 (ABP1) has been described as an important membrane-associated auxin receptor because of a transfer DNA (T-DNA) knockout mutant *abp1-1* as a cornerstone of the study [33]. However, lethal phenotypes observed in *abp1-1* were caused by the deletion of its neighboring gene BELAYASMERT (BSM), not by the knockout of ABP1, thus the *abp1-1* is not a real null allele as described previously [34]. Moreover, another two *abp1* mutants, *abp1-c1* and *abp1-TD1*, did not display any obvious phenotypic defects obtained by CRISPR-Cas9 and T-DNA insertion technology [35]. However, many severe biological defects have been observed in gene knockdown plants [36,37,38]. Therefore, we need to be more cautious about interpreting the results of the experiments using only one simple approach [39]. In some cases, the single null mutant obtained by T-DNA insertion could not separately suppress the dwarfism of *bri1-5* and *bri1-9* mutants. Yet, loss of both homologous genes (*hrd1ahrd1b*, *pawh1pawh2*, *mns4mns5*) leads to the inhibition of the phenotypes of *bri1-5* and *bri1-9* [26,31,40]. The knockout of a gene did not cause any obvious phenotype, which might be thought to have a genetic compensation mechanism [41,42,43,44], but it has not been demonstrated in plants. We recently have discovered a mutant by forward genetics, *sbi3*, which can directly suppress the dwarf phenotypes of *bri1-5*, *bri1-235*, and *bri1-9* [30]. This finding suggests a need for the traditional forward genetic approach to complement the reverse genetics system on gene functional study.

Forward genetics is an effective way to study how misfolded BRI1 is regulated. For instance, *ebs1-7* [8,14,24,25,26,27,28], *sbi1* [29], and *sbi3* [30] are reported to play a vital role in plant development. A variety of *ebs4* mutants can inhibit the phenotype of *bri1-5* and *bri1-9*, meaning that *ebs4* mutants are completely viable mutants [8]. However, a T-DNA insertion might create a null and lethal mutation in *alg12-T* [15]. Interestingly, *ebs4-1* (ag->aa) mutation is presumed to cause abnormal RNA splicing. This can lead to premature termination of translation, thus generating a null allele, yet its mutant phenotype is mild [8]. To make matter worse, *ebs4-2*(S307P) only slightly reduced or impaired the ALG12 activity such that *ebs4-2* was able to rescue the ALG12 activity in the yeast null allele ∆*alg12* cells by recovering the glycosylation defect of CPY, an ER-localized carboxypeptidase Y [8]. Conversely, the *ebs4-3* mutation failed to complement the ∆*alg12* mutants. These results suggest that *ebs4-3* is a possible null allele, but *ebs4-2* is not. Yet, *ebs4-1*, *ebs4-2*, and *ebs4-3* exhibit almost identical plant and other biochemical phenotypes [8]. Thus, it is difficult to reconcile these contradictory findings. To further complicate this problem is that the aforementioned T-DNA knockout *alg12-T* was found to be a lethal allele [15]. After *alg12-T* was crossed to *cce1* (changed calcium elevation 1), another viable *ebs4* allele, they found that calcium response to flg22 treatment in the *cce1 alg12-T* heterozygous line was indistinguishable from the cce1 mutant. This study indicates that *alg12-T* is the real knockout of ALG12 [8,15]. Taken together, it raises the question of whether *ebs4-1* is a true frame-shift null mutant and whether *ebs4-3* contains a mutation in a function of SBI2/ALG12/EBS4 only derived in plants? Furthermore, whether *alg12-T* contains a mutation in another essential gene or whether *alg12-T* is regulated by a novel yet unknown mechanism? Isolating an unquestionably null allele of EBS4/ALG12 can at least partially clarify this problem.

Here, we report the identification of a suppressor of a *bri1* mutant, named *sbi2* (a suppressor of *bri1*), by EMS mutagenesis in the same forward genetic screening that led to the identification of *sbi1* and *sbi3* [29,30]. With a W258Stop mutation, the *sbi2* suppressed the phenotypes of ER-localized *bri1* mutants. *sbi2* had been identified as a true null allele of *EBS4* (EMS-mutagenized *bri1* Suppressor 4), which encodes α-1, 6 mannosyltransferase of specific glycosyltransferases that catalyzes the addition of 8th Man residues to C branch in N-linked glycosylation in *Arabidopsis*. The *sbi2* (W258Stop) mutation significantly altered the growth and development of *Arabidopsis* plants. The point mutation from G1889 to A1889 resulted in the conversion of Try (W) to a stop codon (*) at the 258th position of SBI2/ALG12/EBS4, generating a null mutant. We found that the abundance of BRI1 protein was increased in double mutants of *sbi2 bri1* by inhibiting ER-BRI1 protein degradation. *sbi2* could regulate the ERAD of the ER-localized BRI1-5, BRI1-9, and BRI1-235 through a post-transcriptional mechanism. Moreover, the mutant *sbi2* restored the sensitivity of *bri1-5*, *bri1-9*, and *bri1-235* to BRs. Furthermore, *sbi2* could increase plant sensitivity to ER stress and salt stress from lab environments. Our findings thus provide an insight into the elucidation of the detailed functions of SBI2/ALG12/EBS4 in plant growth and stress response, revealing the necessity to combine forward genetics and reverse genetics together to study gene functions.

## 2. Results

### 2.1. The sbi2 Mutation Partially Suppresses the Phenotypes of bri1-5, bri1-9, and bri1-235

**Figure 1:**
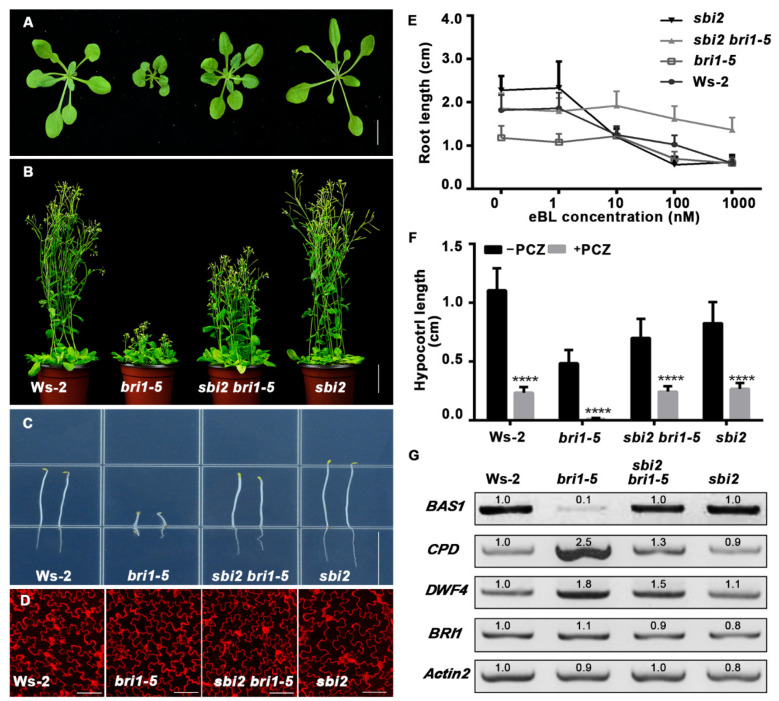
The *sbi2* mutant partly suppressed *bri1-5* phenotypes. (**A**) 3-week-old rosettes of Ws-2, *bri1-5*, *sbi2 bri1-5*, and *sbi2* grown in the soil under a long-day condition (16/8 h, light/dark). Bar: 1 cm. (**B**) Images of 2-month-old mature plants of Ws-2, *bri1-5*, *sbi2 bri1-5*, and *sbi2*. Bar: 3 cm. (**C**) Hypocotyls of 5 days seedlings of Ws-2, *bri1-5*, *sbi2 bri1-5*, and *sbi2* grown on 1/2 MS medium in the dark. Bar: 1.5 cm. (**D**) The morphology of cotyledon pavement cells from 7-day-old seedlings of Ws-2, *bri1-5*, *sbi2 bri1-5*, and *sbi2*. Cotyledons were stained by propidium iodide (PI). Bar: 100 μm. (**E**) The 24-epibrassinolide (eBL)-induced root inhibition assay. 8-day-old seedlings of Ws-2, *bri1-5*, *sbi2 bri1-5* and *sbi2* grown on 1/2 MS medium with different concentrations of eBL under long-day (16/8-h light/dark) condition. Data are means ± SD (standard deviation). N ≥ 30. (**F**) Quantitative analysis of the hypocotyl length of 5-day-old seedlings grown in 1/2 MS medium with or without 5 µM PCZ were plotted as histogram. **** *p* < 0.0001 as two-way ANOVA with Sidak’s multiple comparisons test. (**G**) Semiquantitative PCR analysis of *BAS1*, *CPD*, *DWF4*, and *BRI1* in 2-week-old seedlings of Ws-2, *bri1-5*, *sbi2 bri1-5*, and *sbi2*. BR biosynthetic genes: *CPD* and *DWF4*, a BR inactivation gene *BAS1*. *Actin2* served as an internal control.

The *sbi2* partially suppressed the dwarf phenotype of *bri1-5* at both rosette and mature stages. Morphologically, *sbi2 bri1-5* exhibits a large rosette, a long embryonic stem in the dark, long floral stems at maturity, and a bigger area and perimeter of pavement cells than *bri1-5* mutants (Figure 1A–D and Figure S1). The *sbi2* mutant displayed a similar phenotype to wild-type Ws-2 including narrow leaves with long petioles, rosette size, plant height, and normal fertility under the light conditions. Additionally, the flowering time for *sbi2* was earlier than that of wild-type Ws-2. Fewer numbers of rosette leaves were observed in *sbi2* as well (Figure 1A–D and Figure S2). To determine whether BR responses were altered in the *sbi2* mutants, we used a dose-response assay to examine the effect of 24-epibrassinolide (24-eBL) on root length. We found that *bri1-5* was less sensitive to eBL, but the sensitivity of *sbi2 bri1-5* to eBL was enhanced in a dose-dependent manner. On the other hand, the sensitivity of *sbi2* to BRs was similar to that of wild-type Ws-2 plants (Figure 1E). In addition, we also tested the sensitivity of Ws-2, *bri1-5*, *sbi2 bri1-5*, and *sbi2* to PCZ (Propiconazole, a BR biosynthetic inhibitor), and we found that *bri1-5* was highly sensitive to PCZ, while Ws-2, *sbi2 bri1-5*, and *sbi2* showed less sensitivity to PCZ than *bri1-5* (Figure 1F). Next, we checked the expression level of *DWF4*, *CPD*, and *BAS1* which are known to be reliable markers for BR signaling. In *Arabidopsis*, the *CPD* and *DWF4* are the negative regulators for BRs, while *BAS1* is a positive regulator. When the expression abundance of these BR-responsive genes in plants was analyzed by semiquantitative RT-PCR, similar levels of *BRI1* transcripts were found in Ws-2, *bri1-5*, *sbi2 bri1-5*, and *sbi2* (Figure 1G). Yet, the expression levels of *DWF4* and *CPD* in the wild-type *sbi2* and *sbi2 bri1-5* were significantly downregulated compared to the expression level of *DWF4* and *CPD* in *bri1-5* plants. As expected, compared with *bri1-5*, the expression of *BAS1* in Ws-2, *sbi2*, and *sbi2 bri1-5* was upregulated (G). These results revealed that *sbi2* partially suppressed the phenotypes of *bri1-5*.

**Figure 2:**
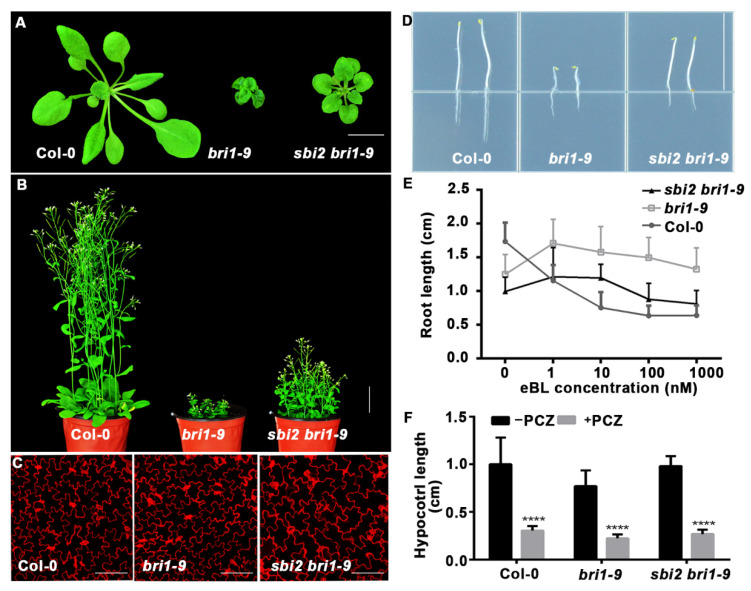
The *sbi2* mutant inhibited the *bri1-9* dwarfism. (**A**) Phenotypes of three-week-old soil-grown seedlings of Col-0, *bri1-9*, and *sbi2 bri1-9*. Bar: 1 cm. (**B**) Pictures of 2-month-old mature plants of Col-0, *bri1-9*, and *sbi2 bri1-9*. Bar: 3 cm. (**C**) Cotyledon pavement cells from 7-day-old seedlings of Col-0, *bri1-9*, and *sbi2 bri1-9*. The seedlings were stained by propidium iodide (PI). Bar: 100 μm. (**D**) Images of 5-day-old dark-grown seedlings of Col-0, *bri1-9*, and *sbi2 bri1-9*. Bar: 1.5 cm. (**E**) The eBL-induced root inhibition assay. Quantitative analysis of root length of 8-day-old seedlings grown in 1/2 MS medium with different 24-eBL concentrations under long-day (16/8-h light/dark) conditions. N ≥ 30 seedlings. Error bar represents ± standard deviation (SD), three independent assays. (**F**) Quantitative analysis of hypocotyl length of 5-day-old seedlings grown in 1/2 MS medium with or without 5 µM PCZ in the dark. **** *p* < 0.0001 as two-way ANOVA with Sidak’s multiple comparisons test.

*bri1-9* is also an ER-localized mutant, carrying Ser 662 Phe mutation and underwent a classic proteasome-dependent ERAD pathway [8]. We suspected that if the *sbi2* could partially suppress the phenotypes of *bri1-5*, it could also suppress *bri1-9* dwarfism. Consistent with our prediction, when crossed into *bri1-9*, *sbi2* resulted in a significant suppression of the *bri1-9* dwarf phenotype and restored the sensitivity of the *bri1-9* to exogenous eBL and PCZ (Figure 2A–F and Figure S1).

**Figure 3:**
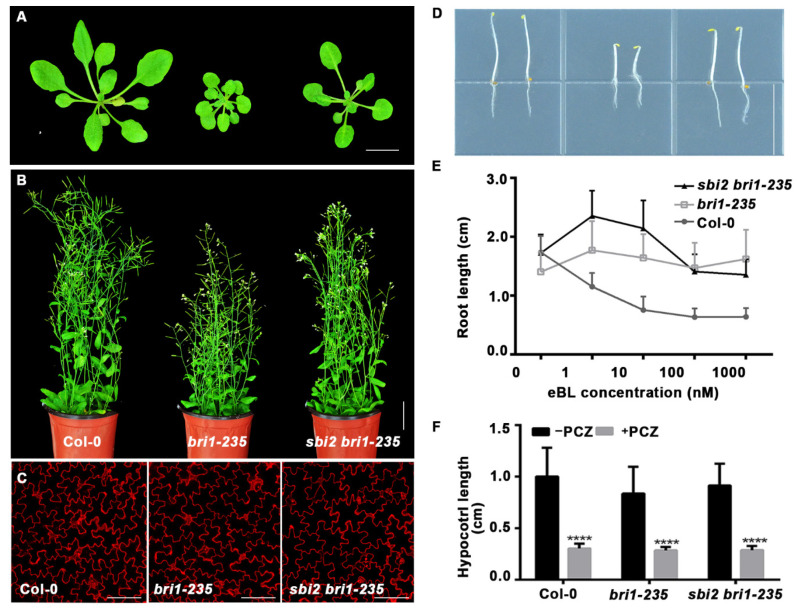
The *sbi2* mutation also suppresses the *bri1-235* dwarfism. (**A**) Phenotypes of 3-week-old soil-grown seedlings of Col-0, *bri1-235*, and *sbi2 bri1-235*. Bar: 1 cm. (**B**) Pictures of 2-month-old mature plants of Col-0, *bri1-235*, and *sbi2 bri1-235.* Bar: 3 cm. (**C**) Cotyledon pavement cells from one-week seedlings of Col-0, *bri1-235*, and *sbi2 bri1-235*. The seedlings were stained by propidium iodide (PI). Bar: 100 μm. (**D**) Col-0, *bri1-235*, and *sbi2 bri1-235* seedlings grown on 1/2 MS medium in the dark for 5 days. Bar: 1.5 cm. (**E**) The eBL-induced root inhibition assay. Quantitative analysis of root length of 8-day-old seedlings grown in 1/2 MS medium with different 24-epibrassinolide (eBL) concentrations under long-day (16/8-h light/dark) conditions. N ≥ 30 seedlings. Error bar represents ± standard deviation (SD). (**F**) Quantitative analysis of hypocotyl length of 5-day-old seedlings grown in half strong MS medium with or without 5 µM PCZ in the dark. **** *p* < 0.0001 as two-way ANOVA with Sidak’s multiple comparisons test.

To further test the role of SBI2/ALG12/EBS4, we also used another ER-localized *bri1* mutant, *bri1-235,* carrying Ser156Phe mutation in the 4th LRR of BRI1′s extracellular domain, and degraded by a proteasome-independent ERAD process [23,30]. Consistently, *sbi2* also suppressed *bri1-235* dwarf phenotypes, including a small rosette, a short hypocotyl, and short stems of mature plants and small perimeter and area of pavement cells. In an eBL-induced root inhibition experiment, we found that the double *sbi2 bri1-235* and *sbi2* mutants inhibited root growth in a dose-dependent manner as compared to *bri1-5*. PCZ treatment showed that the hypocotyl growth of Col-0, *bri1-235*, and *sbi3 bri1-235* seedlings exhibited expected differential sensitivity (Figure 3A–F and Figure S1). Taken together, SBI2/ALG12/EBS4 might regulate ER-localized BRI1.

### 2.2. A Single Base Substitution from G to A Results in a Null Allele of SBI2

**Figure 4:**
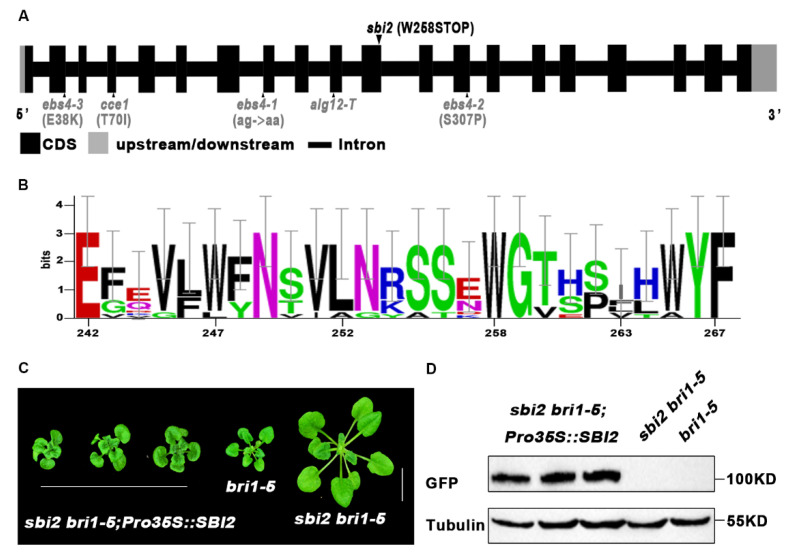
An amino acid substitution from Trp (W) to Stop at the 258th position of SBI2/ALG12/EBS4 in *sbi2*. (**A**) Sequencing analysis of *sbi2* identified a single nucleotide Trp 258 to Stop mutation in At1g02145 (EBS4). (**B**) Sequence alignment of a small part of the SBI2/ALG12/EBS4 protein among different species. W residue at the 258th position was highly conserved. (**C**) Three-week-old soil-grown plants of *bri1-5*, *sbi2 bri1-5*, and three *SBI2*-complemented *sbi2 bri1-5* transgenic lines carrying a *SBI2* transgene driven by the 35S promoter. (**D**) Protein expression levels of *bri1-5, sbi2 bri1-5*, and the corresponding transgenic plants with GFP tag shown in (**C**) were detected with antiGFP antibody. Tubulin served as the loading control.

To identify the sequence alteration that resulted in the *sbi2 bri1-5* phenotype, we compared the genomic DNA sequences of the *sbi2 bri1-5* mutant with the wild-type Ws-2. We found that, apart from the *bri1-5* mutation site (G to A), another single base substitution from G to A was identified in the *EBS4* gene that encodes a α-1, 6 mannosyltransferasea (ALG12) in *Arabidopsis*. This base substitution resulted in a conversion of tryptophan (W) to stop code (*) located at reside 258 of SBI2/ALG12/EBS4 (Figure 4A). Furthermore, a sequence alignment of SBI2/ALG12/EBS4 showed that residue W258 is highly conserved among SBI2/ALG12/EBS4 from different plant species (Figure 4B). The phenotypic analysis of the *sbi2 bri1-5* and the single-base exchange indicated that the mutant resulted from the loss-of-function mutation in the *SBI2/ALG12/EBS4* gene. To confirm our hypothesis, we generated transgenic plants that expressed *SBI2* by introducing a 35S promoter-driven *p35S:SBI2-GFP* construct into *sbi2 bri1-5*. Consistent with our prediction, the independent transgenic lines exhibited shorter petiole as well as rosette width compared to *sbi2 bri1-5* (Figure 4C,D). These results confirmed that *SBI2* completely rescued the phenotype of *sbi2 bri1-5* to near *bri1-5* and that the phenotype of *sbi2* mutant is caused by the loss-of-function mutation in the *SBI2/ALG12/EBS4* gene. Conversely, *p35S:SBI2(1-257)-GFP* could not rescue the phenotype of *sbi2 bri1-5* (Figure S3), suggesting that *sbi2* is a true null allele. Together, we confirmed a role of SBI2/ALG12/EBS4 in the regulation of ER-localized BR receptors.

**Figure 5:**
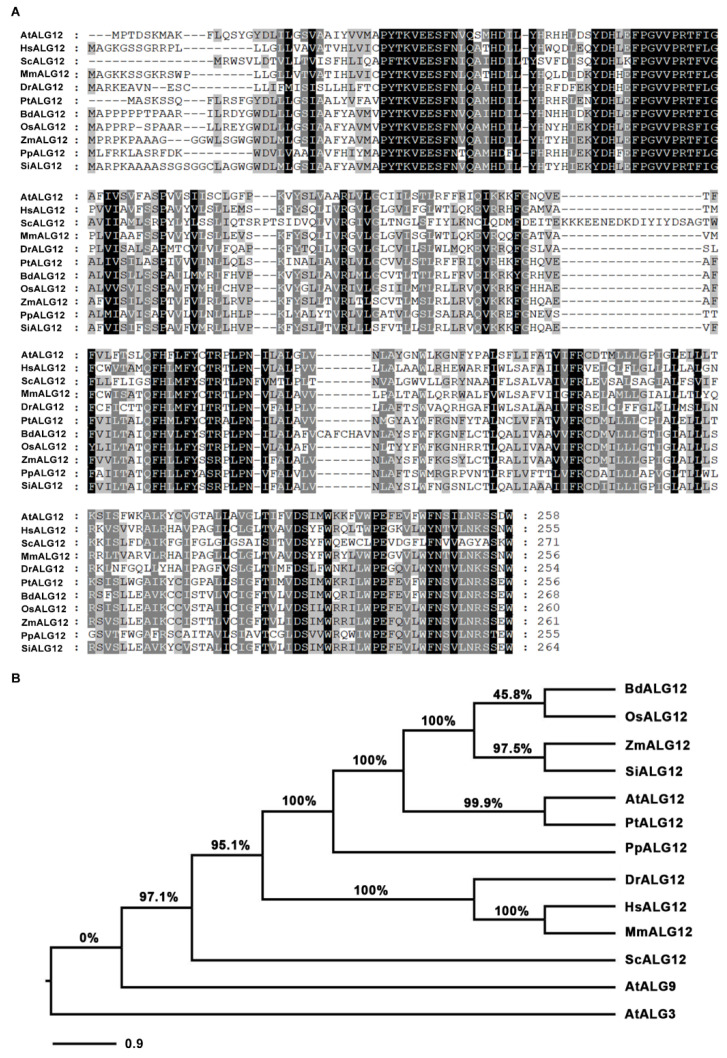
The multiple sequence alignment and phylogenetic analysis. (**A**) The multiple sequence alignment of the amino acid 1-258 domain was performed with MEGA software. The domain is relatively conservative, the 258th residue W is highly conserved. (**B**) The phylogenetic analysis of all species used in (A). *At: Arabidopsis thaliana*, *Hs: Homo sapiens*, *Sc: Saccharomyces cerevisiae*, *Mm: Mus musculus*, *Dr: Danio rerio*, *Pt: Populus trichocarpa*, *Bd:Brachypodium distachyon*, *Os: Oryza sativa*, *Si: Setaria italica*, *Zm: Zea mays*, *Pp: Physcomitrella patens*. (https://www.ncbi.nlm.nih.gov/, accessed on 5 February 2022; https://phytozome-next.jgi.doe.gov/, accessed on 5 February 2022).

Moreover, we also conducted a multiple sequence alignment and a phylogenetic analysis for ALG12 in the selected species. The results showed that there were many conserved domains in the sequence region of 1-258 (Figure 5A). ALG12 in plants are clustered together on the same branch, sister to the animal ALG12 which is polygonal. Although ALG3 (which catalyzes the addition of the 6th mannose) and ALG9 (which catalyzes the addition of the 7th and 9th mannose) are also mannose transferases, they function completely differently from ALG12 (which catalyzes the addition of the 8th mannose), so AtALG3 and AtALG9 could act as an outgroup of the phylogenetic tree (Figure 5B). Furthermore, AtALG12, AtALG3, and AtALG9 were widely expressed in different tissues (Figure S4). The structural model of SBI2/ALG12/EBS4 was predicted by I-TASSER (Figure S5).

### 2.3. The sbi2 Mutation Alters the Abundance and Localization of Glycoprotein

**Figure 6:**
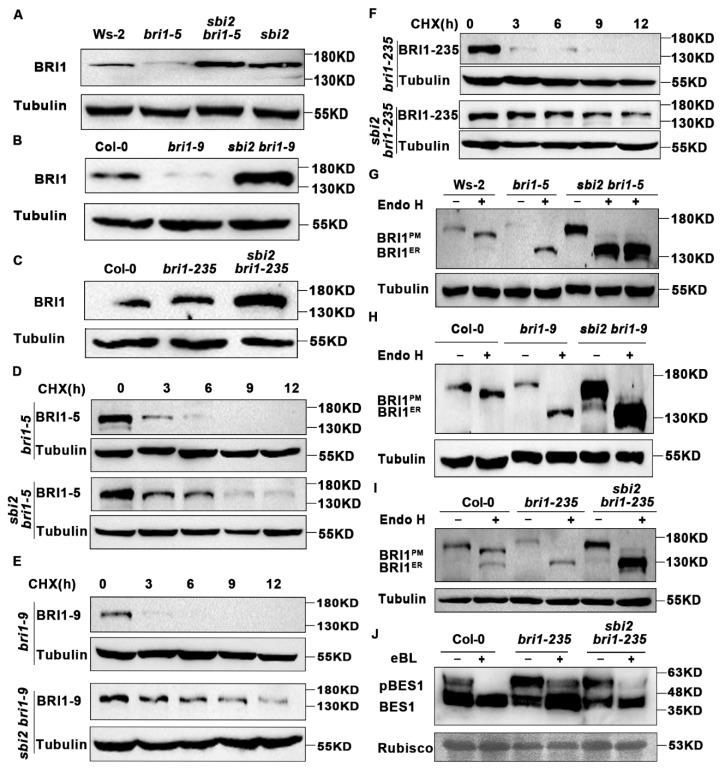
The *sbi2* mutation alters the abundance and localization of the BRI1 protein. (**A**) Western blot analysis of BRI1 protein abundance in Ws-2, *bri1-5*, *sbi2 bri1-5*, and *sbi2*. Tubulin was used as the loading control. (**B**) Western blot analysis of BRI1 protein abundance in Col-0, *bri1-9*, and *sbi2 bri1-9*. (**C**) Western blot analysis of BRI1 protein abundance in Col-0, *bri1-235*, and *sbi2 bri1-235*. Specific antibodies: Anti-BRI1, Anti-Tubulin (control). (**D**) Immunoblot analysis of BRI1-5 stability in *sbi2 bri1-5* with the anti-BRI1 antibody. Two-week-old seedlings were treated with 180 μM CHX for indicated incubation times. (**E**) Immunoblot analysis of BRI1-9 stability in *sbi2 bri1-9* with the anti-BRI1 antibody. Two-week-old seedlings were treated with 180 μM CHX for indicated incubation times. (**F**) Immunoblot analysis of BRI1-235 stability in *sbi2 bri1-235* with the anti-BRI1 antibody. Two-week-old seedlings were treated with 180 μM CHX for indicated incubation times. (**G**) Endo H analysis of 2-week-old seedlings of Ws-2, *bri1-5*, *sbi2 bri1-5*, and *sbi2*. BRI1^PM^ indicates the localization in the plasma membrane (PM) of glycosylated BRI1. BRI1^ER^ indicates the localization of deglycosylated BRI1 in the endoplasmic reticulum (ER). Tubulin was used as the loading control. (**H**) Endo H analysis of 2-week-old seedlings of Col-0, *bri1-9*, and *sbi2 bri1-9*. (**I**) Endo H analysis of 2-week-old seedlings of Col-0, *bri1-235*, and *sbi2 bri1-235*. (**J**) Immunoblotting analysis of 1μM eBL-induced dephosphorylation of Col-0, *bri1-235*, and *sbi2 bri1-235*. Rubisco served as a loading control.

To test whether the observed morphology was caused by either the alteration in abundance of BRI1 protein or by changes in localization of the BRI1 protein, we looked for the abundance of BRI1-5 protein and found that the abundance of BRI1-5 protein was higher in double mutant *sbi2 bri1-5* than in *bri1-5*. Similarly, the abundance of BRI1-9 and BRI1-235 was also increased in the double mutant than in the *bri1-9* and *bri1-235* (Figure 6A–C). To determine whether the increasing abundance of BRI1 was caused by an increase of biosynthesis or a decrease of ERAD of BRI1-5, BRI1-9, and BRI1-235, we treated the *bri1-5*, *bri1-9*, *bri1-235*, and their corresponding double mutants with 180 µM cycloheximide (CHX, a protein biosynthesis inhibitor) (Abcam). As shown in Figure 6D–F, BRI1-5 and BRI1-235 became undetectable after 9 h of CHX treatment, and BRI1-9 became undetectable after 6 h of CHX treatment. In contrast, the mutant BR receptor BRI1 in *sbi2 bri1-5*, *sbi2 bri1-9*, and *sbi2 bri1-235* was quite stable even after 12 h of CHX treatment. This result suggests that the increasing abundance of BRI1 is largely caused by a decrease of degradation rather than an increase of biosynthesis of BRI1-5, BRI1-9, and BRI1-235 (Figure 6D–F). To uncover the detailed mechanism, we next conducted an endoglycosidase H (Endo H) assay, which is an excellent tool for distinguishing the ER-localized protein from the plasma membrane (PM)-localized protein because Endo H can cleave high mannose-type N-glycans in the ER but not the Golgi-processed complex glycan [8]. As shown in Figure 6G–I, BRI1 proteins were still largely retained in the ER in *sbi2 bri1-5*, *sbi2 bri1-9*, and *sbi2 bri1-235*, with a very small quantity of that BRI1 escaped to the cell surface (Figure 6G–I). We next examined the phosphorylation status of transcription factor BES1, which marks the activated BR signaling in dephosphorylated form. The dephosphorylation of BES1 was obviously induced by 1 µM exogenous eBL for 1 h and accumulated more in *sbi2 bri1-235* compared to *bri1-235* (Figure 6J). Altogether, SBI2/ALG12/EBS4 can regulate the ERAD of misfolded glycoproteins through a post-transcriptional mechanism.

### 2.4. The sbi2 Mutation Cannot Regulate the ERAD of PM-Trapped BRI1

**Figure 7:**
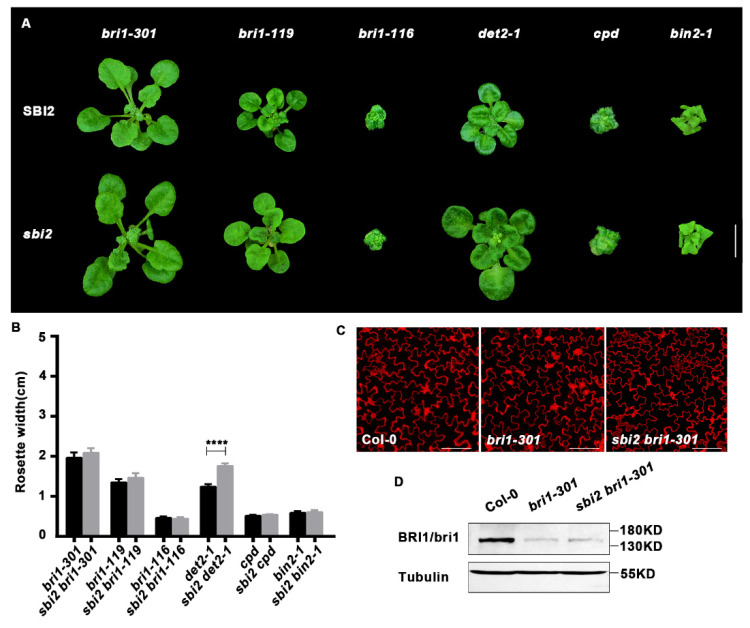
The double mutants of other backgrounds. (**A**) *bri1-301*, *bri1-119*, *bri1-116*, *cpd*, *det2-1*, *bin2-1*, and their corresponding double mutants in *sbi2* background grown in soil for 3 weeks. (**B**) Comparison of rosette width of 3-week-old plants in (**A**). Measurements were performed using the ImageJ software. Data are means ± SD, n ≥ 10. **** *p* < 0.0001 as two-way ANOVA with Tukey’s multiple comparisons test. (**C**) The morphology of cotyledon pavement cells from Col-0, *bri1-301*, and *sbi2 bri1-301*. Cotyledons of 7-day-old seedlings were stained by (PI). Bar: 100 μm. (**D**) Western blotting analysis of BRI1 protein abundance in Col-0, *bri1-301*, and *sbi2 bri1-301*.

To investigate whether *sbi2* monitors only functional or even only ER-specific BRI1 proteins to enhance BR signaling, we crossed *sbi2* to mutants carrying the null *bri1-116* allele, the *bri1-119* allele with a mutation in the extracellular domain of BRI1, or the *bri1-301* allele with a mutation in the kinase domain. We found that *sbi2* did not suppress the phenotypes of *bri1-116*, *bri1-119*, or *bri1-301*. To test whether endogenous BRs were required for *sbi2*-dependent enhancement of BR signaling, we also crossed *sbi2* to *det2-1* and *cpd*, which are a weak and strong mutant in BR biosynthesis, respectively. It was interesting to see that *sbi2* partially rescued the phenotype of *det2-1*, but it could not repress the phenotype of *cpd*. The differentially phenotypic effects of *sbi2* on the *det2-1* and *cpd* mutations suggest the involvement of BRs in numerous processes throughout its life cycle. To address the role of SBI2/ALG12/EBS4 in the BR signaling cascade, we crossed *sbi2* to *bin2-1*, a key negative regulator of BRI1 signaling [29,45]. After crossing, *sbi2 bin2-1* plants were still dwarfed like *bin2-1* plants. Therefore, *sbi2* could not rescue dwarfed phenotype of *bin2-1*. This demonstrates that SBI2 does not activate signaling downstream of BIN2 and that SBI2 may function upstream of BIN2 (Figure 7A–D). Hence, SBI2/ALG12/EBS4 might not regulate the ERAD of PM-trapped BRI1.

### 2.5. The sbi2 Mutation Involved in Plant Resistance to ER and Salt Stress

**Figure 8:**
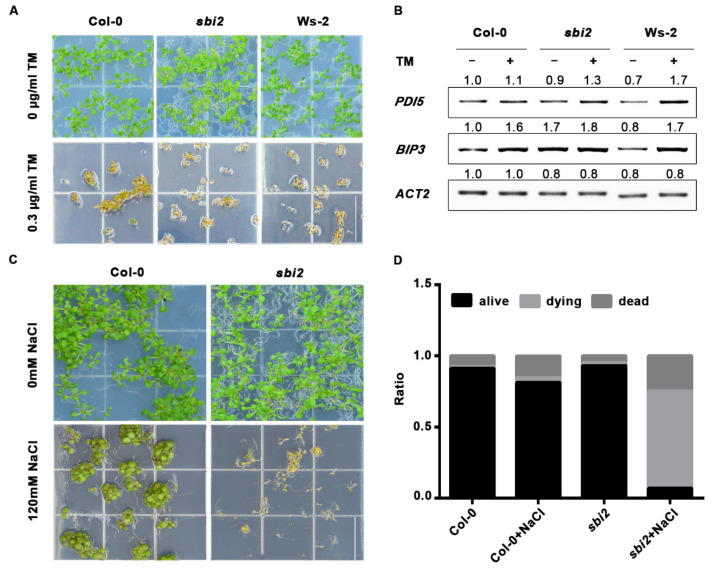
The stress response of *sbi2* to TM and NaCl. (**A**) The photographs of 7-day-old seedlings of wild-type and *sbi2* grown in 1/2 MS with or without 0.3 μg/mL TM. Bar = 1.5 cm. (**B**) Expression levels of *PDI5* and *BIP3* in wild-type and *sbi2* with or without 5 µg/mL TM for 6 h. *ACT2* served as a control. (**C**) The photographs of 12-day-old seedlings of wild-type Col-0 and *sbi2* grown in 1/2 MS with or without 120 mM NaCl. Bar = 1.5 cm. (**D**) The ratio of seedlings in (**C**) was shown in the bar graphs: alive (black), dying (light gray), and dead (dark gray). These experiments were repeated three times.

Previous studies have shown that defective mutations in components of ERQC often lead to the accumulation of abnormal proteins, leading to activation of the unfolded protein response (UPR) pathway, a highly conserved ER stress response pathway [8,30,46]. In this pathway, the ER chaperone responds to the tunicamycin (TM, an ER stress inducer that inhibits protein glycosylation) to maintain protein homeostasis [30]. The previous studies have shown that the binding protein (BIP), protein disulfide isomerase (PDI), and calcein/calconin (CRT/CNXs) were increased in *ebs4-1 bri1-9* and *ebs4-2 bri1-9* compared to WT and *bri1-9* [8]. To test whether *sbi2* mutants affect plant ER stress tolerance, *Arabidopsis* wild-type and *sbi2* seedlings were grown on 1/2 MS medium containing TM 0.3 μg/mL for 7 days. *sbi2* was found to be less tolerant to TM (Figure 8A). RT-PCR analysis showed that the expression level of *BIP3* and *PDI5* was higher in *sbi2* seedlings treated with 5 μg/mL TM for 6 h (Figure 8B). Additionally, we found that *sbi2* was less tolerant to salt (Figure 8C,D).

## 3. Discussion

BR is a crucial polyhydroxylated steroid phytohormone. As the plasma membrane-localized leucine-rich repeat-receptor kinase, BRI1 directly perceives BRs via its extracellular domain to initiate a signaling cascade that modulates the plant growth and development as well as the stress response [46]. After the completion of biosynthesis and glycosylation in the ER, BRI1 must finally reach to its final destination (PM) to function. Mislocalization of BRI1 appears in many *bri1* mutants, such as *bri1-5*, *bri1-9*, and *bri1-235,* which are accompanied by characteristics of misfolded receptor proteins and defects in developmental phenotypes.

In this study, we found that the *sbi2* mutation (W258Stop in SBI2/ALG12/EBS4) partially repressed the dwarfed phenotypes of ER-localized *bri1* mutants, such as *bri1-5*, *bri1-9*, and *bri1-235*, and affected the growth of the wild-type *Arabidopsis*. *SBI2/ALG12/EBS4* encodes a α1, 6 mannosyltransferase of specific glycosyltransferases in N-linked glycosylation in *Arabidopsis*. Thus, a possible reason is that *sbi2* mutation made ER-localized proteins, BRI1-5, BRI1-9, or BRI1-235 become overaccumulated by a saturated ER retention machinery. Indeed, the Endo H assay indicates that *sbi2* mutation allows a small quantity of BRI1-5, BRI1-9, or BRI1-235 proteins to escape from the ER to the cell surface to function there as normal BR receptors (Figure 6). We further uncovered that the increasing receptor proteins are largely caused by inhibiting protein degradation rather than increasing the abundance of proteins by biosynthesis. As such, *sbi2* mutation suppresses the *bri1-5*, *bri1-9*, or *bri1-235* mutant phenotypes by altering the localization and quantity of the receptor proteins. Another possible reason is that reducing the numbers of Man residues of N-glycans on *bri1-5*, *bri1-9*, or *bri1-235* might reduce the affinity of the receptors to interact with ER lectins. A lot of cell surface receptors are decorated with multiple N-linked glycans in plants. BRI1, as a BR receptor normally localized in the PM to function, should not be an exception. Thus, the *sbi2* mutation might affect the glycosylation of BRI1 in the ER with a reduced activity. Meanwhile, sequence analysis indicates that this acidic residue is located within the highly conserved region. Moreover, ALG12 is also highly conserved among plants. ALG12 may catalyze the addition of 8th Man residues in the assembly of Dol-PP-Glc_3_Man_9_GlcNAc_2_. When exogenous TM and NaCl were used to mimic ER stress and salt stress, respectively, *sbi2* reduced plant stress tolerance. In fact, stressful conditions do accentuate the accumulation of additional impaired and misfolded proteins in the ER in *sbi2* mutants (Figure 8).

N-linked glycosylation is a complex process. During N-glycosylation, mutations in most enzymes result in a defect of assembly or of its subsequent transfer process. Further investigation by overexpression of *SBI2/ALG12/EBS4* in *ebs3-1 bri1-9* shows an α1,6 Man added to the N-glycan precursor, revealing that the glycan signal marks ERAD clients BRI1-9, which promotes the ERAD of BRI1-9. Hence, *SBI2/ALG12/EBS4* -overexpressing transgenic lines in *ebs3-1 bri1-9* have more severe growth phenotypes [14]. Therefore, SBI2/ALG12/EBS4 is likely very important in the regulation of the ERAD of misfolded proteins.

In *Arabidopsis thaliana*, *ebs4* mutants isolated by forward genetics were most healthy as weak alleles. Further analysis of the mutational sites together with the characteristics of *ebs4* mutants reveals a profound complication (Figure S6) [8]. Firstly, *ebs4-2*(S307P) only slightly reduced or impaired the ALG12 activity [8]. Secondly, a changed calcium elevation 1 mutant (*cce1*: T70I) and *ebs4-3* (E38K) were located in a highly conserved long loop facing the ER lumen [8,15]. Thirdly, *ebs4-1* (ag->aa) mutation is suggested to cause abnormal RNA splicing and lead to premature termination of translation [8]. Yet, it has not confirmed by cNDA sequencing. Finally, *EBS4* expression rescued several biochemical defects of the *ebs4* mutations (*ebs4-2 bri1-9* and *ebs4-3 bri1-5*), suggesting that they are all loss-of-function mutants. However, when *EBS4*, *ebs4-2*, and *ebs4-3* were individually transformed into the yeast ∆*alg12* cells to test whether the *EBS4* gene can complement the function of the yeast ALG12, the *Arabidopsis* wild-type *EBS4* and *ebs4-2* were able to rescue the ALG12 activity in yeast cells, in which the glycosylation defect of CPY, an ER-localized carboxypeptidase Y was remedied. Conversely, the *ebs4-3* mutation failed to complement the ∆*alg12* mutation, revealing that *ebs4-2* might only slightly reduce the ALG12 activity in yeast cells. Interestingly, the *ebs4-2* mutant exhibits almost identical phenotypes to *ebs4-1*. It is difficult to comprehend these contradictory results on the CPY glycosylation pattern and the similar phenotypes in *ebs4* mutants. By contrast, the aforementioned T-DNA knockout *alg12-T* was lethal [15]. After *alg12-T* was crossed with *cce1* (changed calcium elevation 1), it was found that calcium response to flg22 treatment in the *cce1 alg12-T* heterozygous line was indistinguishable from the *cce1* mutant. This study indicates that *alg12-T* was a real knockout of ALG12 [15]. Here, we found that *sbi2*, a real null allele inhibited the ERAD of ER-trapped BRI1-5, BRI1-9, and BRI1-235. Taken together, these results may demonstrate that *sbi2*, a true null allele, is viable, in favor of the findings from the study of *ebs4* mutants isolated by the forward genetic approach but against the study by the T-DNA knockout, highlighting a lethality problem in genetics.

On the other side of the problem is the genetic redundancy. We have recently reported an extreme example, in which single T-DNA knockout mutants *mns4-1* and *mns5-1* cannot suppress the dwarf phenotypes of *bri1-5* and *bri1-9* but only their double mutants *mns4-1 mns5-1* can [31], while we showed that *sbi3*, a loss-of-function allele of *MNS5* can sufficiently inhibit the phenotypes of *bri1-5*, *bri1-9*, and *bri1-235* [30]. Together with the repetitive isolation of viable loss-of-function alleles *ebs4/sbi2/cce1* by forward genetics but a lethal allele *alg12-T* by T-DNA knockout in SBI2/ALG12/EBS4, our thoroughly forward genetic study clearly advocates the necessity of using both forward genetics and reverse genetics on the study of the functional genes [30,39].

## 4. Materials and Methods

### 4.1. Plant Materials and Growth Conditions

The ecotype Columbia (Col-0) and Wassileskija-2 (Ws-2) of *Arabidopsis thaliana* was used as wild-type control. The mutant *bri1-5* was in Ws-2 background, *bri1-9*, *bri1-235*, *bri1-116*, *bri1-301*, *det2-1*, *cpd*, and *bin2-1* were in the Col-0 background, the mutant *bri1-119* was in Enkheim-2 (En-2). The *bri1-5 sbi2* was identified as a suppressor for *bri1-5* by mutagenized 0.4% ethyl methane sulfonate (EMS) (Sigma-Aldrich). The *sbi2*, *bri1-235 sbi2*, and *bri1-301 sbi2* were selected by crossing *bri1-5 sbi2* and Ws-2 *bri1-235* and *bri1-301*, respectively. Seed sterilization and plant growth conditions were described previously [23]. The sensitivity of root and hypocotyl to exogenous 24-epibrassinolide (24-eBL, Sigma) or propiconazole (PCZ, a brassinosteroid biosynthesis inhibitor) (Solarbio) were performed as previously described [30]. The seedlings were grown vertically on media plates for 7 days, and around 20-30 plants were used to measure the hypocotyls and roots from each plate.

### 4.2. Plasmid Construction and Generation of Transgenic Plants

To complement the *sbi2 bri1-5* mutant, the *SBI2/ALG12/EBS4* coding region was amplified from cDNA of the WT using gene-specific primers (*SBI2* SacⅠ F: CGAGCTCATGCCGACGGATTCGAAAATG, *SBI2* BamHⅠ R: CGGATCCACATCCAGGCCATTTCTTAT). The amplified fragment was first cloned into a T-Vector, PMD19, and then introduced into the SacⅠ- and BamHⅠ-digested binary vector, pCHF3, that carries the 35S promoter and a green fluorescent protein (GFP) to get p35S:*SBI2*-GFP construct. These binary vectors were transformed into Agrobacterium tumefaciens, strain GV3101, followed by plant (*sbi2 bri1-5*) transformation with the floral dip method (Clough and Bent, 1998). Transgenic lines were selected on 1/2 MS medium containing 50 mg/L kanamycin.

### 4.3. Protein Extraction and Endo H Treatment

The seedlings from agar with or without endoglycosidase H (Endo H) (New England Biolabs) or leaves from 2-week-old soil-grown plants were homogenized, boiled, and centrifuged. After centrifugation, the protein extracts were denatured at 100 °C for 10 min in denaturing buffer and incubated with or without Endo H in the G5 buffer for 1 h at 37 °C. The immunoprecipitated proteins were separated by 8% SDS/PAGE and immunoblotted with anti-BRI1 antibody (Agrisera). Horseradish peroxidase-linked antirabbit antibodies were used as secondary antibodies, and the signal was detected by Western blotting. The experiments were repeated three times.

### 4.4. Transcript Analysis by RT-PCR

The seedlings of wild-type and mutants from 1/2 MS plates were collected. Total RNAs were extracted by using RNeasy Plant Mini Kit (Qiagen). The mRNA concentration was estimated by spectrophotometer. First-strand cDNA was synthesized from 2 mg of total RNA by utilizing M-MLV, a reverse transcriptase (Invitrogen), following the manufacturer’s instruction. The resulting cDNAs corresponding to 100 ng of total RNAs were amplified by using Step One Plus Real-Time PCR System (Applied Biosystems) with gene specific primers for *DWF4*, *CPD*, *BAS1*, and *Actin2* at an annealing temperature of 55–58 °C for 26–30 cycles [47]. RT-PCR experiment was repeated three times. Primers used for RT-PCR are listed in Table S1.

### 4.5. Observation on Pavement Cell

One-week-old seedlings were stained in 10 µg mL^−1^ propidium iodide (PI, Sigma) for 10 min and washed three times in deionized water (10 min/ time). The pavement cells of cotyledons were observed by laser scanning confocal microscope (Leica TCS SP8). Images were obtained on condition of laser excitation at 561 nm with emission at 590–630 nm. The lobe length, neck width, perimeter, and area were measured by Image J. Circularity was analyzed [30,48].

### 4.6. Stress Treatment on ER and Salt Stress

The seeds of *Arabidopsis* were germinated and grew on half-strength MS medium with or without 0.3 µg/mL tunicamycin (TM, Abcam) for 7 days. Two-week seedlings were treated with 5 µg/mL TM for 6 h or with 0.25% DMSO (control) for RT-PCR analysis. The seeds were germinated and grew on half-strength MS medium with 120 mM NaCl for 12 days to cause stress response.

## Data Availability

All datasets generated for these findings are available in the main text and the Supplementary Materials, further inquiries can be directed to the corresponding author.

## Supplementary Materials

The following supporting information can be downloaded at: https://www.mdpi.com/1422-0067/23/10/5811/s1.
