# Supplementary material for: The Role of SBI2/ALG12/EBS4 in the Regulation of Endoplasmic Reticulum-Associated Degradation (ERAD) Studied by a Null Allele
Source: Int J Mol Sci. 2022 May 22;23(10):5811. doi: 10.3390/ijms23105811 (PMC9147235; doi:10.3390/ijms23105811)
Supplement: Supplementary file 1 [file ijms-23-05811-s001.zip › ijms-1725017-supplementary.pdf]

The Role of SBI2/ALG12/EBS4 in the Regulation of Endoplasmic Reticulum-Associated Degradation (ERAD) Studied by a Null Allele

Xiaoxia Sun, Di Zhao, Khawar Ali, Yumeng Zhu, Guang Wu * and Guishuang Li *

	Ws-2	*bri1-5*	*sbi2 bri1-5*	*sbi2*	Col-0	*bri1-9*	*sbi2 bri1-9*	*bri1-235*	*sbi2 bri1-235*
Lobe (μm)	18.29±0.387	11.24±0.236 ****	14.19±0.224 ***	17.68±0.335	19.55±0.297	13.29±0.269 ****	14.08±0.339	14.4±0.322 ****	14.46±0.279
Neck (μm)	17.08±0.296	20.1±0.34 ****	19.25±0.283	18.62±0.312	15±0.146	17.15±0.424 ****	18.11±0.288	19.22±0.323 ****	18.11±0.388
Perimeter (μm)	578.8±15.78	381.3±12.87 ****	455.6±14.31 *	619.8±16.6	492.9±12.38	308.9±13.7 ****	358.8±14.29	407.1±12.58 ***	453.2±13.27
Area (μm^2)	5181±153.4	4101±171.6 ****	4761±203.7	5271±76.45	4687±116.3	2621±142.7 ****	3558±152.7 ***	3930±153.4 **	4035±129.6
Circularity	0.199±0.007	0.361±0.011 ****	0.292±0.009 ***	0.179±0.008	0.251±0.006	0.356±0.012 ****	0.362±0.015	0.302±0.007 **	0.256±0.012

Figure S1. The size of cotyledon pavement cells. The measurements of cotyledon pavement cells were performed using ImageJ software. N $\geqslant$ 30 seedlings for measurements. * $p < 0.05$, ** $p < 0.01$, *** $p < 0.001$, **** $p < 0.0001$ as Tukey's multiple comparisons test.

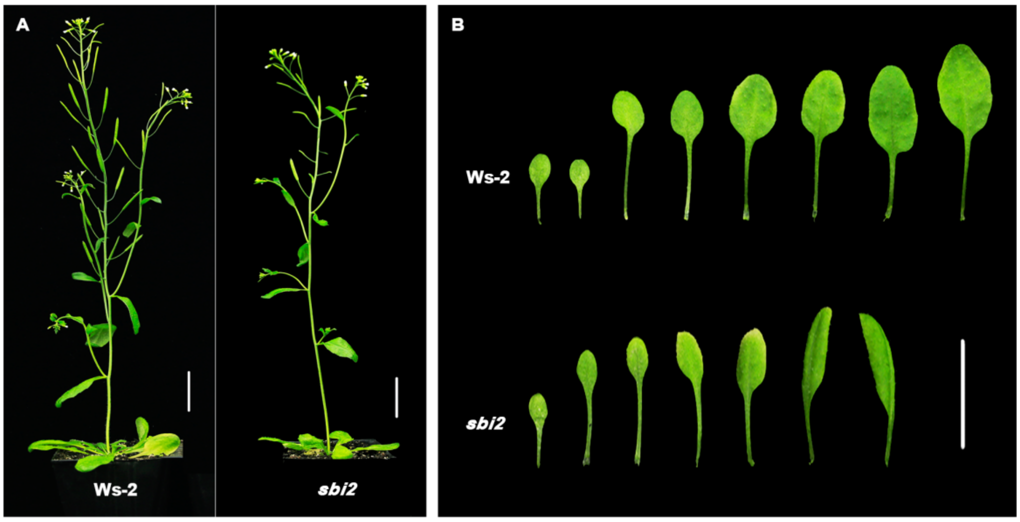

Figure S2. Phenotypes of *Arabidopsis* wild type Ws-2 and mutant *sbi2*. (**A**) The comparison of 2-month-old mature plants of Ws-2 and *sbi2*. Scale bar = 3 cm. (**B**) Fewer leaves were exhibited in *sbi2* than Ws-2. Scale bar = 2 cm.

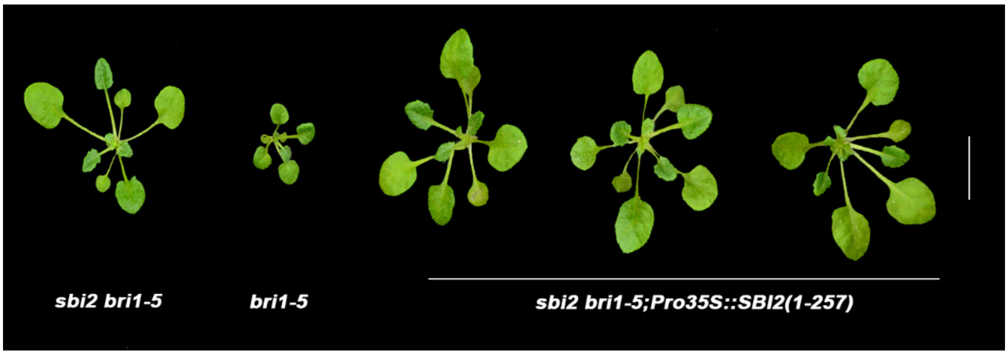

Figure S3. Three weeks soil-grown plants of *sbi2 bri1-5*, *bri1-5*, three transgenic lines carrying a *SBI2(1-257)* transgene driven by the 35S promoter. Scale bar = 1 cm.

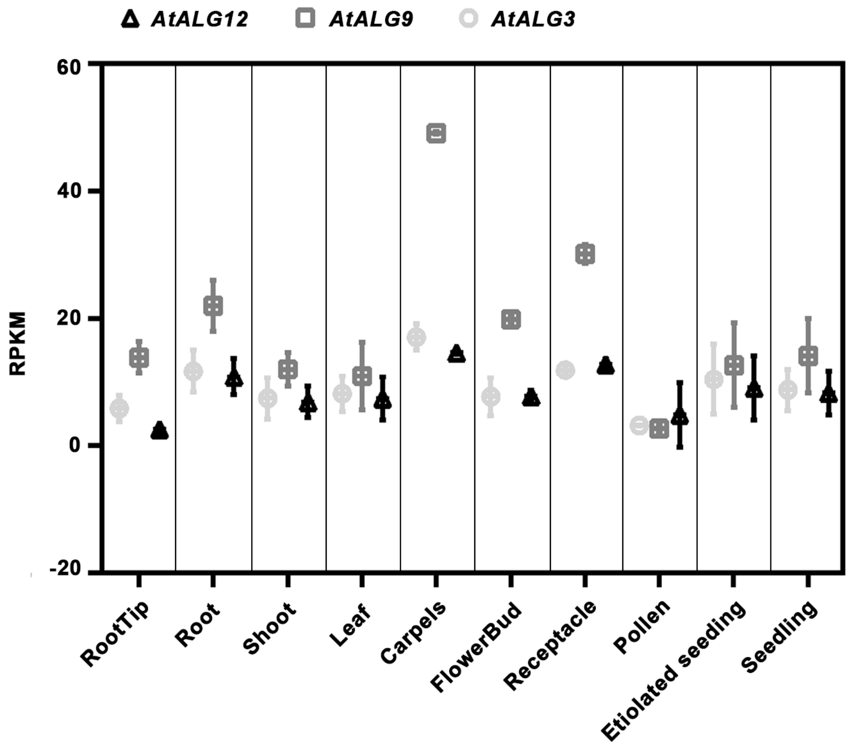

Figure S4. Expression levels of *AtALG12*, *AtALG3* and *AtALG9* were plotted as graph in different tissues (https://bar.utoronto.ca/eFP-Seq_Browser/).

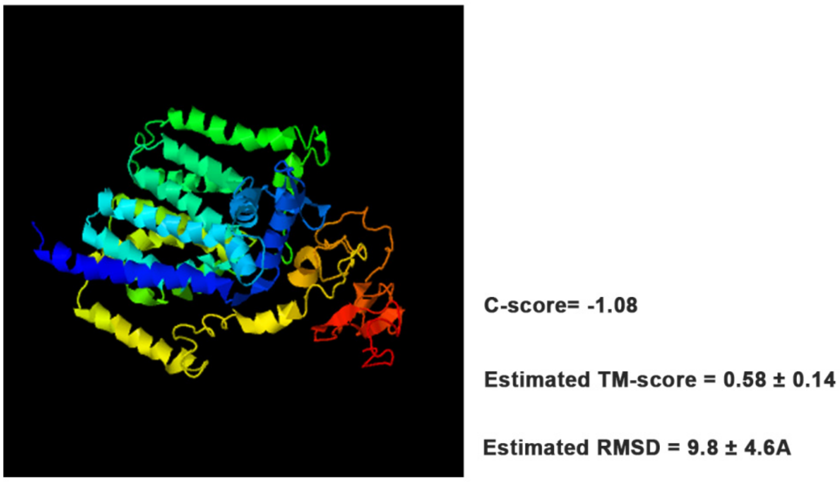

Figure S5. The structure model of SBI2/EBS4 was predicted by I-TASSER. score is in -5, 2 and C-score > -1.5 indicates a model of correct global topology. (https://zhanglab.ccmb.med.umich.edu/I-TASSER/).

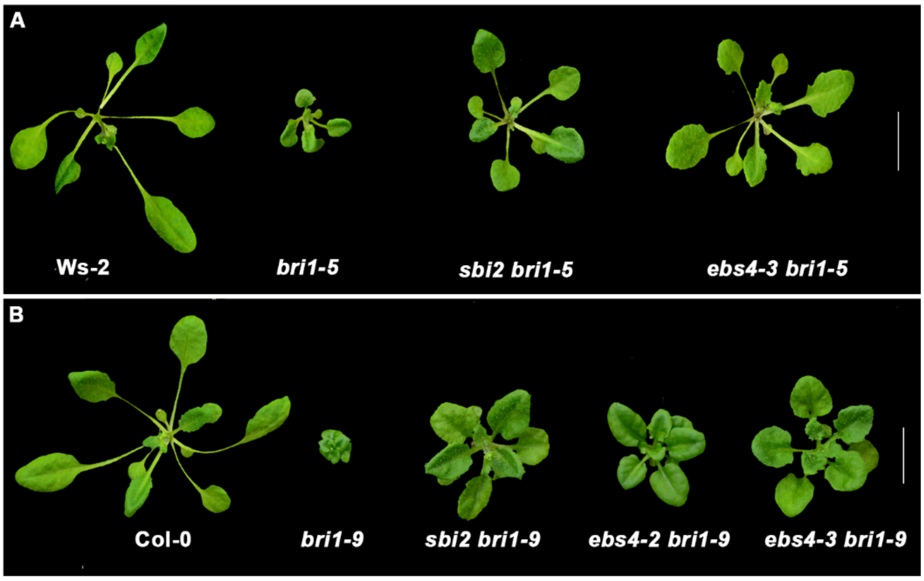

Figure S6. 3-week-old seedlings grown in the soil under a long-day condition (16/8 h, light/dark). (**A**) Phenotypes of three-week-old soil-grown seedlings of Ws-2, *bri1-5, sbi2 bri1-5*, and *ebs4-3 bri1-5.* Scale bar, 1 cm. (**B**) Phenotypes of three-week-old seedlings of Col-0, *bri1-9*, *sbi2 bri1-9*, *ebs4-2 bri1-9* and *ebs4-3 bri1-9*. Scale bar, 1 cm.

Table S1. Primers used in this study.

Name	Sequence (5′-3′)
Actin2-RT-F	ACTCTCCCGCTATGTATGTCG
Actin2-RT-R	TGGACCTGCCTCATCATACTC
BRI1-RT-F	GGTAGAGAGATGAGGAAGAGA
BRI1-RT-R	CTCATAATTTTCCTTCAGGAACTTC
CPD-RT-F	GTTCTTATCCTGCTTCCATTTG
CPD-RT-R	AGCCACTCGTAGCGTCTCATT
DWF4-RT-F	CGAAGGAAGGCTCTTTGAATG
DWF4-RT-R	CTTCAACGGCTTTAGGGCAA
BAS1-RT-F	GTTCAGGACATTGTGGAGGAG
BAS1-RT-R	GGATAAAGCAACATAAGGACG
SBI2/EBS4Sac1-F	CGAGCTCATGCCGACGGATTCGAAAATG
SBI2/EBS4BamH1-R	CGGATCCACATCCAGGCCATTTCTTAT
BiP3-RT-F	TTCGAAGTGGACGCAAATGG
BiP3-RT-R	CTATAGCTAACCGACTCAAAAGACCT
PDI5-RT-F	CTCGTGAAGCTGAGGGTATTG
PDI5-RT-R	TGTGCGAAATCTAACTCAGAG
ACT2-RT-F	GGTAACATTGTGCTCAGTGGTGG
ACT2-RT-R	AACGACCTTAATCTTCATGCTGC
bri1-5-genotyping-F	AGAATCAAGAGATAGGTGGTTGGG
bri1-5-genotyping-R	GCTGGTTAAAGAAGCAGAGCA
bri1-9-genotyping-F	TAACAATGGTTCGATGATGTTTCTGGACAAGC
bri1-9-genotyping-R	GCATAGTAAGAGCTGACATAGCCTG
bri1-235-genotyping-F	TCTCAACGTCGGATTCAGTGC
bri1-235-genotyping-R	AAGATGTTGCAGAGCAGAGC
bri1-301-genotyping-F	CATCGAAATCTTGTGCCTCTTCTTG
bri1-301-genotyping-R	CTCGGGGTCAAACACATCGCTAATC
bri1-119-genotyping-F	ACCCTTGTAATATCACTAGCAGAGTCTATGAAG
bri1-119-genotyping-R	CGATTTCCGTAAGCATAGTAAGAGC
bri1-116-genotyping-F	TGGCGAGTTACCGATGGATACG
bri1-116-genotyping-R	CTCTTAGATCACCTACCTCATCAGG
det2-1-genotyping-F	AGGATGACTACGAAGACGGAAAC
det2-1-genotyping-R	CAGCCCAGCCCAACCACTCAATAAGCT
LBb1.3	ATTTTGCCGATTTCGGAAC
cpd-22-LP	TTTCTTTCTCTCCGCTCCTTC
cpd-22-RP	CTACTCCGCCGTACACGTTAC
