# Supplementary figures and images for: The Role of SBI2/ALG12/EBS4 in the Regulation of Endoplasmic Reticulum-Associated Degradation (ERAD) Studied by a Null Allele
Source: Int J Mol Sci. 2022 May 22;23(10):5811. doi: 10.3390/ijms23105811 (PMC9147235; doi:10.3390/ijms23105811)

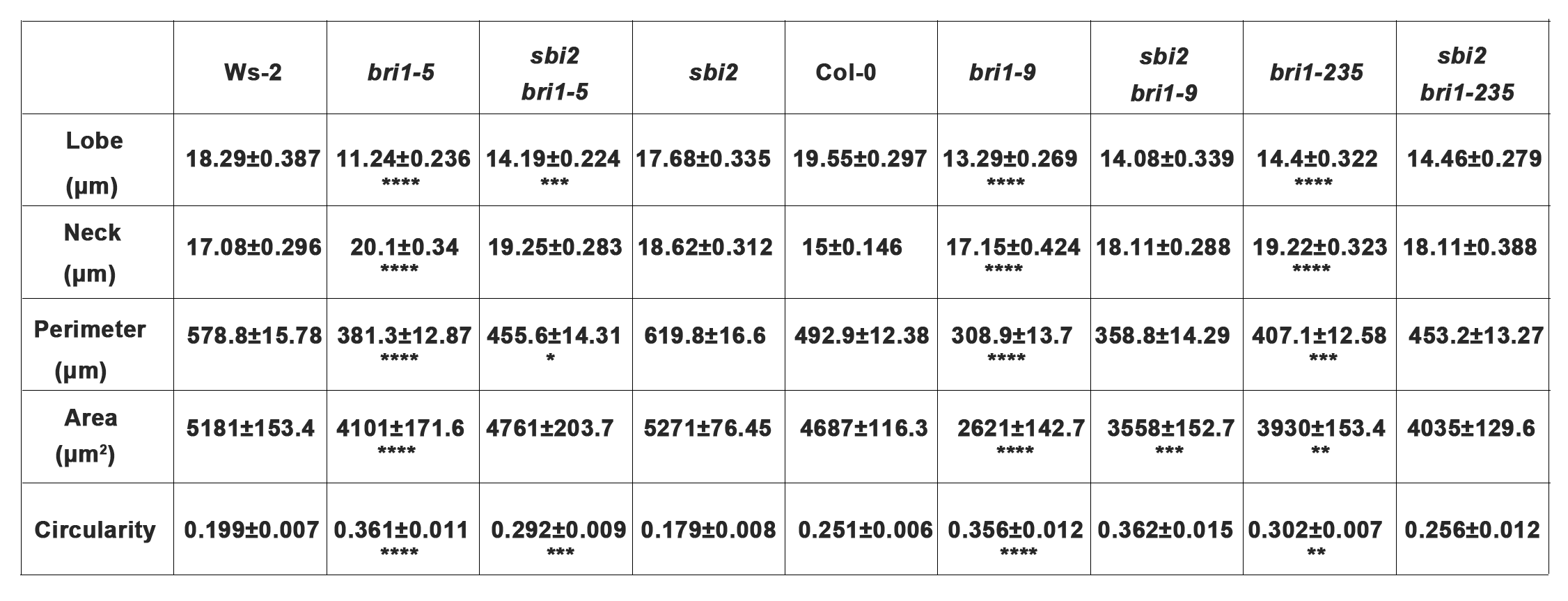

Supplement: Supplementary file 1 [file ijms-23-05811-s001.zip › Figture S1.tif]

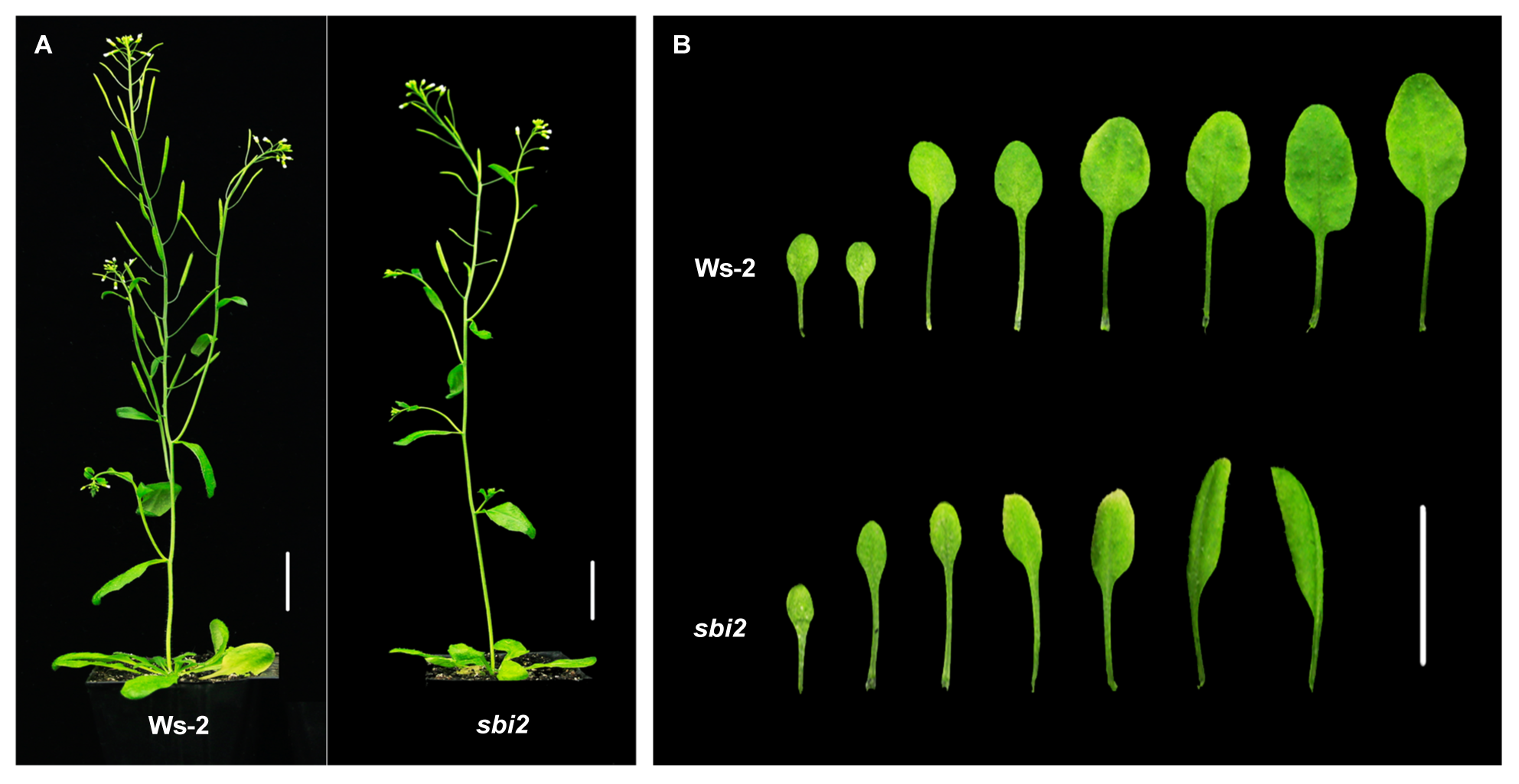

Supplement: Supplementary file 1 [file ijms-23-05811-s001.zip › Figture S2.tif]

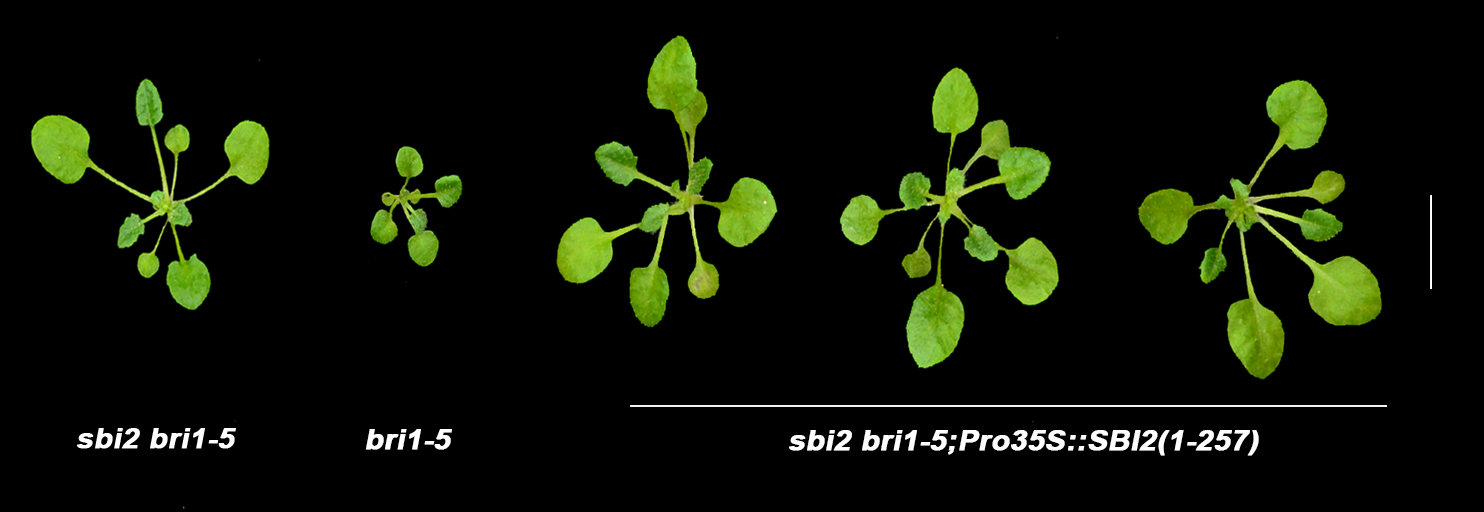

Supplement: Supplementary file 1 [file ijms-23-05811-s001.zip › Figture S3.tif]

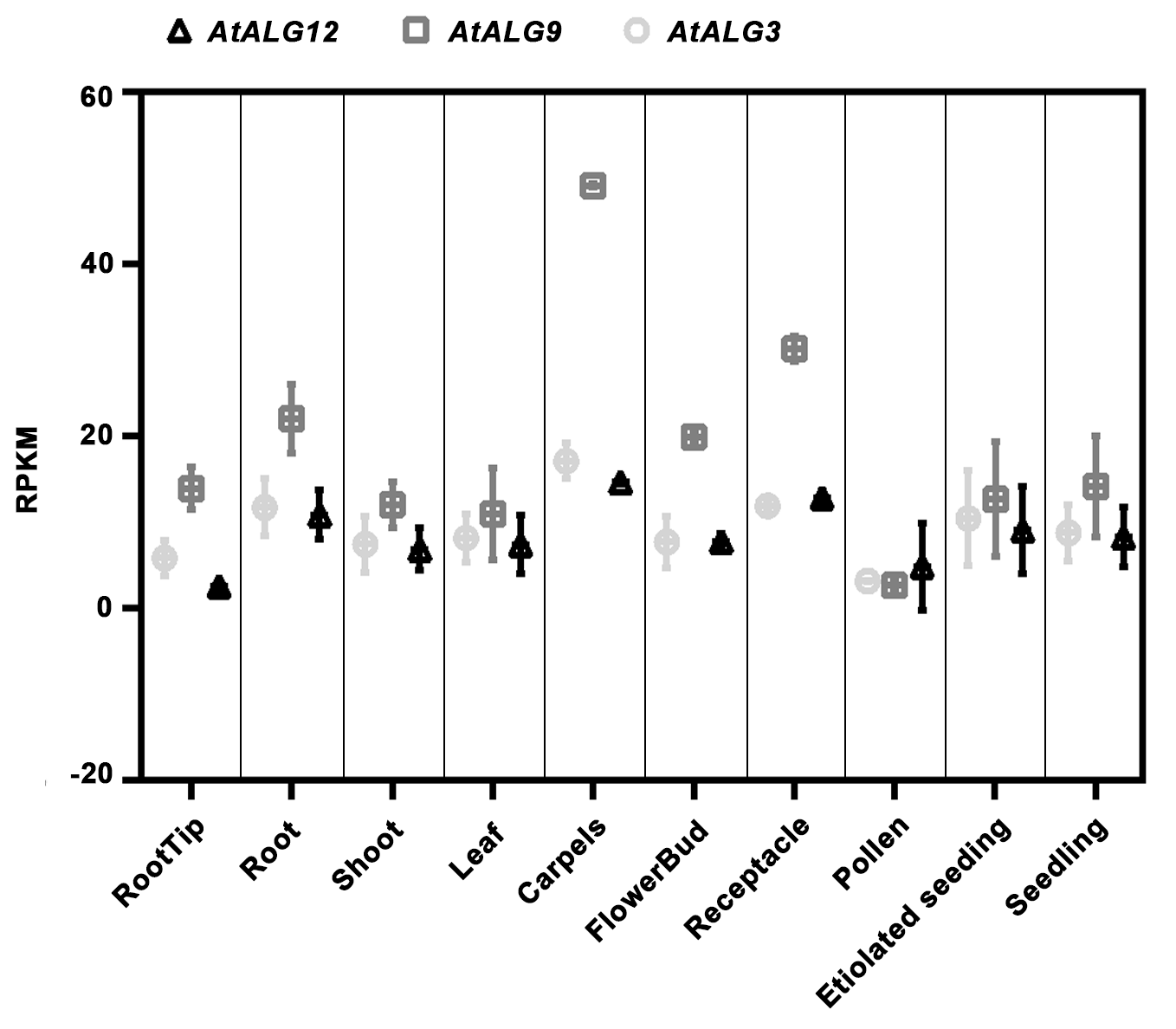

Supplement: Supplementary file 1 [file ijms-23-05811-s001.zip › Figture S4.tif]

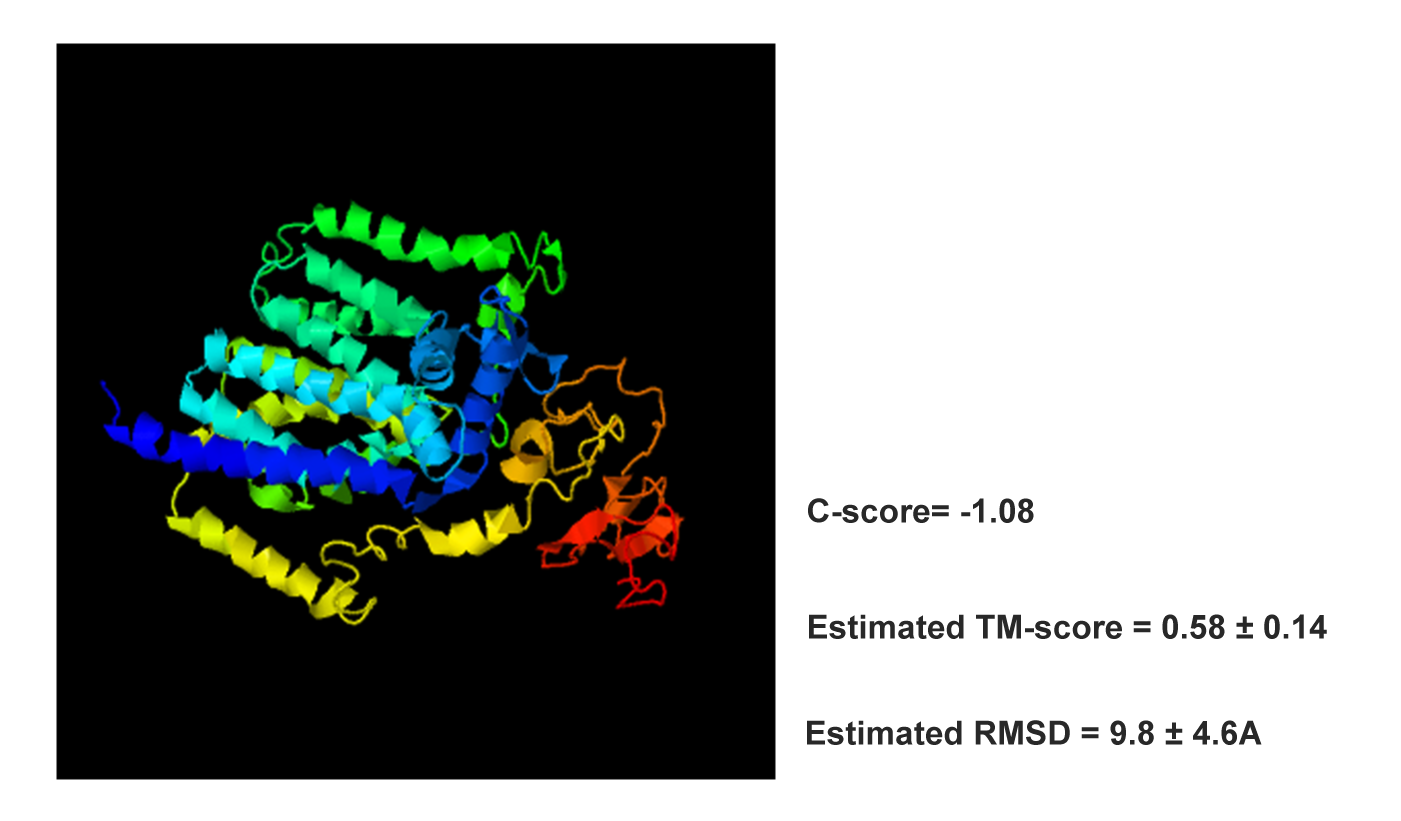

Supplement: Supplementary file 1 [file ijms-23-05811-s001.zip › Figture S5.tif]

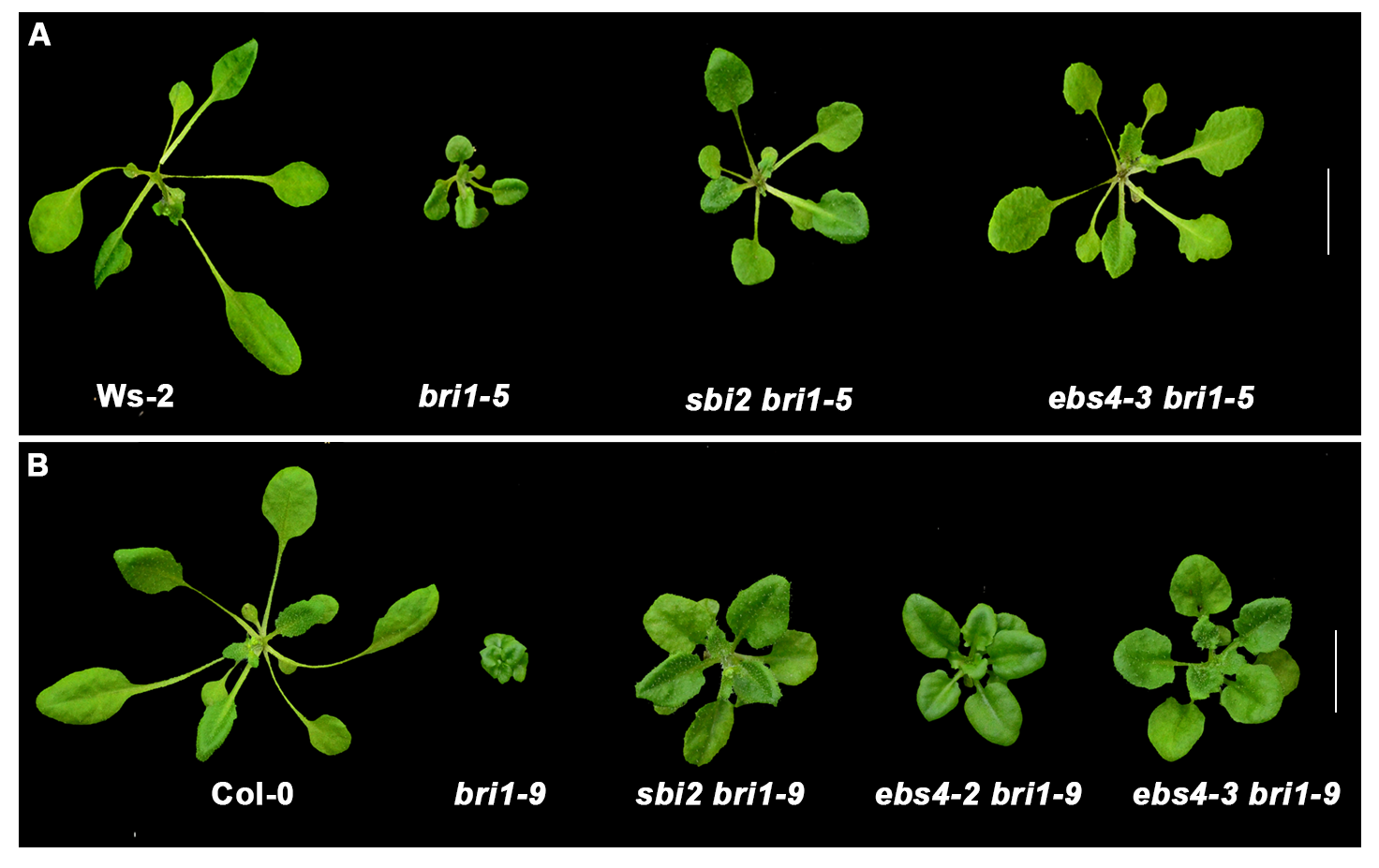

Supplement: Supplementary file 1 [file ijms-23-05811-s001.zip › Figture S6.tif]
